# Use of a Novel Whole Blood Separation and Transport Device for Targeted and Untargeted Proteomics

**DOI:** 10.3390/biomedicines12102318

**Published:** 2024-10-11

**Authors:** Colin T. McDowell, Amanda L. Weaver, Nylev Vargas-Cruz, Nathan K. Kaiser, Charles M. Nichols, Gary A. Pestano

**Affiliations:** Biodesix Inc., 919 W. Dillon Rd, Louisville, CO 80027, USA; colin.mcdowell@biodesix.com (C.T.M.); amanda.weaver@biodesix.com (A.L.W.); nylev.vargas@biodesix.com (N.V.-C.);

**Keywords:** plasma proteomics, mass spectrometry, lateral flow, LC-MS/MS

## Abstract

Background: There is significant interest in developing alternatives to traditional blood transportation and separation methods, which often require centrifugation and cold storage to preserve specimen integrity. Here we provide new performance findings that characterize a novel device that separates whole blood via lateral flow then dries the isolated components for room temperature storage and transport. Methods: Untargeted proteomics was performed on non-small cell lung cancer (NSCLC) and normal healthy plasma applied to the device or prepared neat. Results: Significantly, proteomic profiles from the storage device were more reproducible than from neat plasma. Proteins depleted or absent in the device preparation were shown to be absorbed onto the device membrane through largely hydrophilic interactions. Use of the device did not impact proteins relevant to an NSCLC clinical immune classifier. The device was also evaluated for use in targeted proteomics experiments using multiple-reaction monitoring (MRM) mass spectrometry. Intra-specimen detection intensity for protein targets between neat and device preparations showed a strong correlation, and device variation was comparable to the neat after normalization. Inter-specimen measurements between the device and neat preparations were also highly concordant. Conclusions: These studies demonstrate that the lateral flow device is a viable blood separation and transportation tool for untargeted and targeted proteomics applications.

## 1. Introduction

Liquid biopsies are utilized for clinical testing in personalized medicine applications, including informing physicians on effective treatment strategies and monitoring therapeutic responses to treatment [1]. For protein biomarker discovery and clinical diagnostics, blood is the preferred biospecimen matrix as it is minimally invasive and reflects disease-related biology [2]. However, the process of shipping and handling whole blood prior to analysis can compromise analyte stability, especially for proteins. The fractionation of blood into serum or plasma requires centrifugation of whole blood and manual separation of the liquid fraction from the cellular pellet at the collection site. This is followed by cold storage and transport, often on dry ice, which can be prohibitively complex and costly. There are also inherent biohazards and strict shipping requirements to manage temperature control, as well as the need for specialized equipment such as centrifuges and calibrated temperature shipping monitors. Therefore, there is a desire in the field of proteomics for blood storage and transport methods that do not require centrifugation or cold storage, which may also minimize risks to the specimens and transporters.

An alternative approach for blood storage and transport is dried blood spot (DBS) cards, in which whole blood is applied to filter paper and allowed to dry. The filter is then extracted and processed to separate the cellular from liquid fractions for downstream analysis. DBS biospecimens are generated, stored and transported at ambient temperatures, with some analytes being stable for several months [3]. The advantages of DBS enable biospecimens to be collected remotely from a clinical setting for patient convenience and compliance. In addition, many blood-borne viruses are deactivated when dried, so fewer biohazards are associated with DBS cards compared to handling whole blood [4]. The DBS method offers an attractive approach to efficiently process and analyze patient specimens using mass spectrometry (MS) [5]. DBS cards have been previously shown to be applicable for MS analysis of small molecule therapeutics, drug monitoring, toxicology and metabolic diagnostics, as well as other forensic and veterinary uses [4,6,7]. The simplicity of using DBS cards for small molecule analysis has brought about a heightened interest in modifying this approach for larger biomolecules to provide specimens suitable for proteomic MS analysis [8,9,10,11]. However, as erythrocytes represent ~40–50% of blood, the use of DBS cards can be hindered by undesirable hemolysis that complicates plasma proteomic analyses [12]. The variability in plasma protein abundances between patient specimens, particularly in hemoglobin, creates a major hurdle for proteomic analysis of DBS cards by mass spectrometry [13,14,15,16]. As a result, additional development and characterization of DBS methodologies is required to enable the transfer of a serum or plasma liquid-based MS assay to a DBS approach.

As many clinical tests are conducted with the liquid fraction of whole blood, there has been significant progress in developing blood storage and transport devices that separate the blood into cellular and plasma fractions while retaining the advantages of DBS cards for sample collection, storage and transport [17,18,19,20,21]. This paper further characterizes a device which accepts whole blood and, by principles of lateral flow, separates the cellular from liquid components across a glass fiber membrane, minimizes cell lyses and generates a plasma fraction for downstream analysis [19]. The device provides an alternative to DBS cards by producing dried plasma that stabilizes the analytes of interest and more closely resembles the composition of plasma generated by traditional centrifugation methods. This blood collection device (BCD) has been previously validated in a clinical proteomic matrix-assisted laser desorption ionization time-of-flight (MALDI-TOF) MS-based immune classifier that measures the levels of acute phase immunoinflammatory reactants in non-small cell lung cancer (NSCLC) patient blood [19,20,21]. The test assesses the abundance of circulating serum amyloid A1 (SAA1), serum amyloid A2 (SAA2) and C-reactive protein (CRP) in addition to other proteins as a measure of immuno-inflammation in NSCLC patients [22]. Patients with a sustained inflammatory response, as defined by elevated levels of SAA1, SAA2 and CRP, are classified as NSCLC “Poor” and do not respond to single-agent immunotherapy as well as patients without elevated SAA1, SAA2 and CRP (NSCLC “Good”) [23,24]. “Poor” patients are therefore more likely to benefit from the addition of chemotherapy to their immune checkpoint inhibitor (ICI) regimen.

In this study, the ability of the BCD to support a wider range of proteomic analyses was investigated. First, we studied the use of the BCD for discovery proteomic applications, including how the device membrane affects the detectable plasma proteome. Here the device was used in conjunction with a nanoparticle enrichment workflow to profile the plasma proteome at sufficient depth. We then explored the performance of the BCD with a highly multiplexed, quantitative multiple-reaction monitoring (MRM) MS assay using a panel of isotopically labeled peptide standards derived from 125 plasma proteins with abundances spanning 4–5 orders of magnitude [25]. The results from both sets of experiments demonstrate that the device is a robust and reproducible tool for plasma proteomics, offering the broader clinical proteomic community a safe, low-cost and simple-to-use blood separation solution that eliminates the complexities associated with traditional blood separation, transport and storage protocols.

## 2. Materials and Methods

### 2.1. Samples

In preparation for the discovery proteomics experiments, aliquots of previously drawn plasma from NSCLC patients classified as immune classifier-labeled “Good” and immune classifier-labeled “Poor” were obtained from commercial biobanks under their consent protocols (Discovery Life Sciences, Huntsville, AL, USA; ProMedDx, Norton, MA, USA; ProteoGenex, Inglewood, CA, USA; and BioIVT, Westbury, NY, USA) [22]. These plasma specimens were combined to create “Good” and “Poor” pools for analysis. A normal healthy (NH) donor plasma pool was run as a control and consisted of healthy donors that had been consented under the Biodesix HOPE study (Pro00056631). Blood from these donors was drawn by venipuncture in K_2_EDTA tubes, centrifuged, manually separated and then the plasma was pooled and frozen prior to analysis.

Two hundred and fifty (250) μL aliquots of the NH, “Good” and “Poor” plasma pools were thawed on ice, then applied to the BCD using a single-use pipette. The plasma was allowed to penetrate the device glass fiber membrane for 2 min before the outer housing was sealed and placed in a desiccated, moisture-tight envelope to dry overnight at ambient temperature. A separate 250 μL aliquot from each pool was removed and stored overnight at 4 °C to serve as the “neat” preparation (without application to the BCD). After overnight drying, the device membranes were removed, shredded and placed in 2 mL Eppendorf tubes. The shredded membranes were rehydrated with 750 μL of HPLC-grade H_2_O, leaving 250 μL of supernatant after absorption into the membrane. The samples were vortexed gently for 3 min, then the protein-containing eluate was removed and placed in a new tube. Both the BCD eluates and neat plasma aliquots were then stored on ice to await further processing.

For the targeted proteomic experiments, whole blood was obtained using venipuncture by a licensed medical specialist. Five (5) normal healthy donors consented under the Biodesix protocol prior to any study activity (Pro00056631). Blood was drawn into 3 mL K_2_EDTA blood collection tubes (Greiner Bio-One, Kremsmünster, Austria), which were then gently inverted to distribute the anticoagulant. Whole blood was applied to the device using a 250 μL single-use pipette, where it was allowed to separate into liquid and cellular components by lateral flow for 2 min before being sealed in a desiccated, moisture-tight envelope overnight. Separately, the same blood tubes were then centrifuged at 1800× *g* for 10 min and a 10 μL aliquot of the separated plasma was removed and stored at 4 °C. After overnight drying, a 1.5 × 1.7 cm section was excised from each BCD membrane approximately 0.5 cm from the red blood cell (RBC) separation front. These sections were then quartered and placed into 0.45 µm centrifugal spin filters (VWR, Radnor, PA, USA), where 80 µL of PBS was added to each and they were vortexed for 5 min. The tubes were then centrifuged at 12,000× *g* for 2 min and the eluate was collected. The device eluates and neat plasma aliquots were then stored on ice until analysis.

### 2.2. Proteomic Preparation

Prior to untargeted proteomics, the NH, “Good” and “Poor” preparations, both with the BCD and neat, were enriched for low abundance proteins and processed to peptides using a Proteograph XT assay kit on the SP100 Automation Instrument (Seer, Redwood City, CA, USA). Briefly, 250 μL sample aliquots, along with kit consumables and lab hardware, were loaded onto the instrument deck. Nanoparticle enrichment, tryptic digestion and peptide clean-up were then performed in a 96-well plate format. During the automated method, each sample was split into two wells and incubated separately with each of two nanoparticle mixtures. After clean-up, the concentration of resultant peptides was measured using the Pierce Quantitative Fluorometric Peptide Assay (Thermo Scientific, Waltham, MA, USA). Both calibration curve generation and sample handling were automated using the SP100 instrument. Following preparation, fluorescence measurements were obtained using a SpectroMax M5 Plate Reader (Molecular Devices, San Jose, CA, USA). The peptide concentration of each sample was determined using the standard curve. Post-quantitation, peptides were dried by vacuum centrifugation using a Speedvac Concentrator (Thermo Scientific, Waltham, MA, USA) and stored at −80 °C until reconstitution for LC-MS/MS analysis.

Targeted proteomic preparation was performed with the 125-protein PeptiQuant Plus Proteomic Kit (MRM Proteomics, Montreal, QC, Canada), which uses spiked-in heavy isotope-labeled peptide standards (SIS) for quantitation by multiple-reaction monitoring (MRM) mass spectrometry. Briefly, the samples (10 µL plasma or 15 µL BCD eluate) were reduced by the addition of 300 mM Tris (pH 8.0), 9 M urea, and 20 mM DTT (20 µL for plasma or 30 µL for BCD eluates) and incubated for 30 min at 37 °C. Samples were then alkylated with 20 µL of 100 mM iodoacetamide for 30 min in the dark at ambient temperature. The samples were diluted to 0.5 M urea with 100 mM Tris (pH 8.0) (272 µL for plasma or 440 µL for BCD eluates), followed by the addition of 35 µL of 1 mg/mL tosyl phenylalanyl chloromethyl ketone (TPCK)-treated trypsin in 25 mM Tris (pH 8.0) (Worthington Biochemical Corp., Lakewood, NJ, USA). Samples were then incubated for 18 h at 37 °C on an orbital shaker (250 RPMs). The digest was quenched by acidifying the samples to a final concentration of 1% FA in 700 µL H_2_O.

The acidified digests were then desalted in a 96-well C18 solid-phase extraction (SPE) plate. Forty (40) µL of liquid plasma digest, or 100 µL of BCD digest, was mixed with 10 µL of the SIS peptide mixture resuspended in 30% ACN, 0.1% FA, and 10 µL of 30% ACN, 0.1% FA. Samples were then diluted to a final volume of 600 µL with aqueous 0.1% FA. The SPE plate was conditioned with 500 µL of 90% ACN, 10% H_2_O, and 0.1% TFA and equilibrated with 750 µL of 2% ACN, 98% H_2_O, and 0.1% TFA. A 510 µL aliquot of each sample was then loaded onto separate C18 cartridges in the 96-well plate. The columns were washed once with 500 mL of 2% ACN, 98% H_2_O, and 0.1% TFA and eluted with two rounds of 100 mL of 48% ACN, 52% H_2_O, and 1% TFA. Eluted peptides were then dried overnight in a SpeedVac Concentrator (Thermo Scientific, Waltham, MA, USA) and stored at −80 °C until reconstitution for MRM LC-MS/MS analysis.

### 2.3. Untargeted LC-MS/MS

In preparation for chromatographic separation, all samples were cleaned up once more by SPE using the EvoTip Pure (EvoSep, Odense, Denmark) workflow. Briefly, EvoTip disposable trap columns were washed with 50 μL solvent B (0.1% FA in ACN), conditioned by submersion in 100 μL 2-propanol and washed with 50 μL solvent A (0.1% FA in H_2_O) with centrifugation at 700× *g* for 1 min between all steps. A total of 200 ng of each sample (20 μL of reconstituted peptides at 10 ng/μL) was loaded onto the EvoTips and centrifuged at 700× *g* for 1 min. All samples and blanks were washed with 50 μL solvent A, centrifuged again and stored in 100 μL solvent A. At the time of analysis, prepared samples were loaded onto the deck of the EvoSep One (EvoSep, Odense, Denmark) nanoflow liquid chromatography (nLC) system for injection into the MS. Peptides were separated onto a 15 cm EvoSep Endurance C18 column (150 μm ID, 1.9 μm particle size) using EvoSep’s 30 samples per day (44 min gradient) method with 0.1% FA in H_2_O mobile phase A and 0.1% FA in ACN mobile phase B.

For detection of enriched plasma peptides, an in-house data-independent acquisition (DIA) parallel accumulation serial fragmentation (PASEF) method was used on a timsTOF flex MS fitted with a CaptiveSpray ion source (Bruker Daltonics, Billerica, MA, USA). The instrument was operated in positive mode (100–1700 *m*/*z*, 0.6–1.55 1/K_0_) with a ramp time of 100 ms and an accumulation time of 100 ms, resulting in a 100% duty cycle at a 9.43 Hz ramp rate. Capillary voltage was set to 1600 V and dry gas was pumped at 3.0 L/min with a drying temperature of 200 °C. DIA-PASEF MS/MS parameters were set to acquire 20 mass steps, each with a mass width of 50.00 Da, a 1.00 Da mass overlap and no mobility overlap, resulting in a 0.95 s total cycle time.

### 2.4. Targeted LC-MS/MS

Prior to MRM-MS, samples were reconstituted with 34 µL of 0.1% FA in LC-MS H_2_O. An Agilent 1290 HPLC in-line with an Agilent 6490 triple quadrupole (QQQ) MS was used for the MRM analysis. Ten (10) µL aliquots of the resolubilized digests were injected onto a Zorbax Eclipse Plus RP-UPLC column (2.1 × 150 mm, 1.8 µm particle diameter, Agilent Inc., Santa Clara, CA, USA) at 50 °C with a flow rate of 0.4 mL/min over a 60 min LC method using LC-MS-grade solvents. The column was initially equilibrated with 98% mobile phase A (0.1% FA in H_2_O) and 2% mobile phase B (0.1% FA in ACN). Peptides were eluted using the following gradient: 2% B, increasing to 7% B at 2 min, 30% B at 50 min, 45% B at 53 min, 80% B from 53.5 to 55.5 min and 2% B at 56 to 60 min for column equilibration. The retention time, optimized collision energy and MRM transition for each SIS peptide standard were predetermined and provided by the manufacturer (MRM Proteomics, Montreal, QC, Canada). The MS was programmed to perform MRM analysis on each heavy and light peptide pair at the appropriate retention time using a detection window ranging from 90–120 s, with an MS cycle time of 700 ms. A single transition per peptide target was acquired for quantitation.

### 2.5. Data Analysis

Raw MS data files from the untargeted experiments were imported into Proteograph Analysis Suite (PAS) software (version 3.0.0.3, Seer, Redwood City, CA, USA) for processing. Data files were linked to specific plates, then the samples were searched against the DIA-NN neural network (build 1.8.1) for proteoform ID and relative quantification (Supplemental Data) [26]. For group comparisons, raw MS intensity values were median normalized on a run-by-run basis and missing values were imputed with random values from a normal distribution using a down-shifted mean and fractional standard deviation. All statistical comparisons were made by *t*-test. The resulting graphs were generated in PAS and GraphPad Prism software (version 10.2.3, Dotmatics, Boston, MA, USA). To provide molecular and biological context to the data, gene ontology (GO) enrichment analyses for biological processes, molecular functions and cellular compartments were performed. Protein groups were searched against a reference human proteome and enriched descriptors were determined by the protein analysis through evolutionary relationships (PANTHER) test for overrepresentation using annotations from the GO database (DOI: 10.5281/zenodo.7942786, accessed on 2 April 2024) [27,28,29]. Protein physiochemical properties were manually annotated using the Expasy ProtParam tool (Swiss Institute of Bioinformatics, Lausanne, Switzerland) [30].

Targeted MRM-MS data files were analyzed with Skyline Quantitative Analysis software (Supplemental Data) (version 20.2.0.343, University of Washington) [31]. Calibration curves were generated with known light to heavy peptide ratios using 1/×2 weighted linear regression and used to calculate the protein concentration in fmol/µL as well as the lower limit of quantitation (LLOQ). A single surrogate tryptic peptide was used to establish the endogenous plasma protein concentration. Of the 125 plasma proteins in the panel, 26 were below the limit of quantitation for the normal healthy donors used in the study, 4 did not yield results for one or more samples due to shifted retention time, and 6 had a significantly high %CV (>30%) in both the neat plasma and BCD eluate. Following the removal of those proteins from further analysis, we then analyzed 89 proteins for quantitative protein comparison between the BCD eluate and plasma as well as in the protein gradient investigation.

## 3. Results

### 3.1. Untargeted Proteomics with the Blood Collection Device

**Figure 1:**
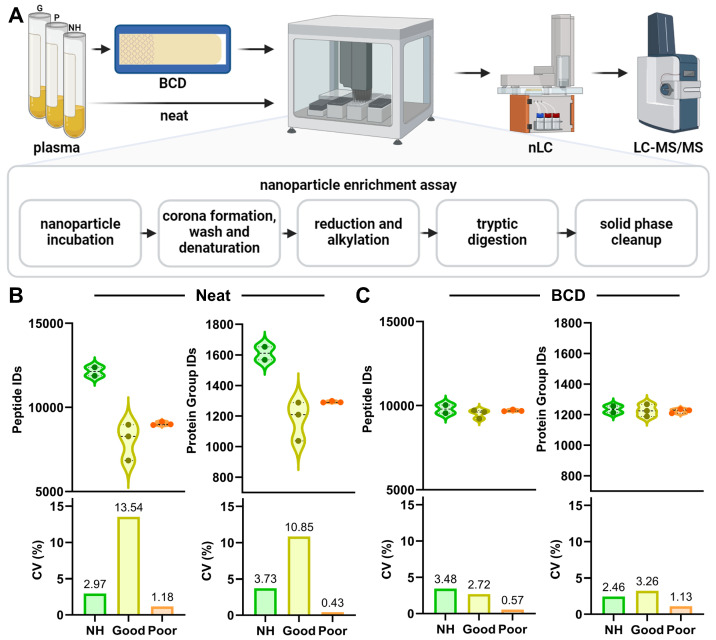
Application of plasma to the BCD reduces variability in protein group identification. (**A**) Overview workflow of neat and device sample preparation for discovery proteomics, including nanoparticle enrichment, enzymatic digestion, chromatographic separation and MS analysis. Violin plots and associated CV measurements for peptide and protein group identifications in (**B**) Neat and (**C**) BCD preparations of the normal healthy (NH, green), NSCLC “Good” (yellow) and NSCLC “Poor” (orange) plasma pools are shown. BCD, blood collection device.

The goal of the study was to characterize the use of the BCD for a broad range of proteomic applications; thus, an initial step was to better define how the device affects the detectable plasma proteome. To this end, we performed discovery proteomic experiments where normal healthy, NSCLC immune classifier-labeled “Poor” and NSCLC immune classifier-labeled “Good” plasma pools were applied to the device then compared to the same pools prepared neat without the use of the BCD. Here we chose a nanoparticle enrichment approach for the proteomic preparation to deplete the sample of the high abundance plasma proteins that often obfuscate protein-level biology differences between healthy and diseased patient plasma [32]. Enriched proteins were then processed to peptides and analyzed by nLC-MS/MS. A general outline of the workflow, with a special focus on nanoparticle enrichment, is described in Figure 1A.

An average of 1611, 1178 and 1293 protein groups from 12,136, 8031 and 9035 peptides were identified in normal healthy (NH, n = 2), NSCLC “Poor” (n = 3) and NSCLC “Good” (n = 3) plasma, respectively, prepared neat without the device (Figure 1B). The percent coefficient of variation (CV) for these identifications ranged from 0.43–13.54% in the neat plasma preparations, where the NSCLC “Good” plasma pool had the highest variability. When applied to the device, an average of 1235, 1227 and 1224 protein groups from 9799, 9525 and 9711 peptides were identified in NH, NSCLC “Good” and NSCLC “Poor” plasma pools, respectively (Figure 1C). Variability in these samples ranged from 0.57–3.48% CV. Overall, the use of the BCD reduced intra-pool variability between technical replicates in all three plasma pools.

### 3.2. Effect of the BCD on the Plasma Proteome

**Figure 2:**
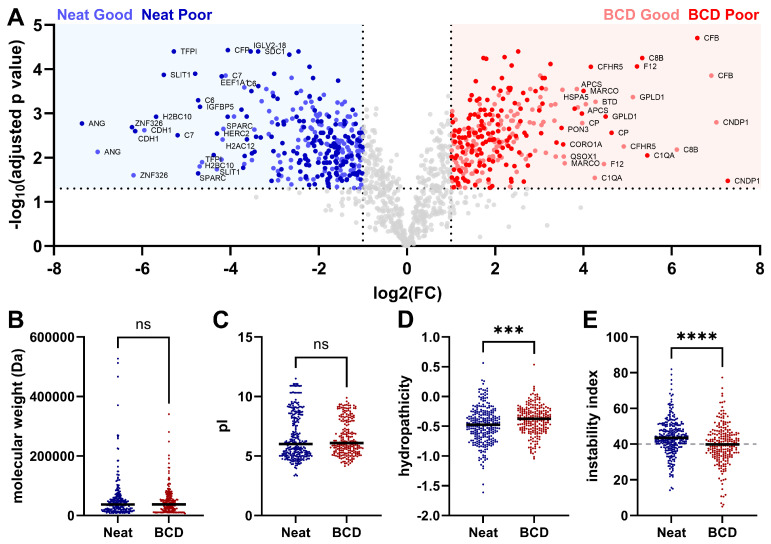
Differential enrichment of plasma proteoforms between the BCD and neat preparations in immune classifier-stratified plasma pools. (**A**) Overlaid volcano plots of “Good” and “Poor” NSCLC plasma prepared neat and on the device (“Good” on device, light red; “Good” neat, light blue; “Poor” on device, dark red; “Poor” neat, dark blue; log_2_ FC > 1, −log_10_ adjusted *p* value > 1.3 [<0.05]). Physiochemical characterization of neat- (blue) and BCD-associated (red) protein groups, including (**B**) molecular weight, (**C**) pI, (**D**) hydropathicity and (**E**) instability index (stable:unstable demarcation of 40 marked in gray). FC, fold change; ns, not significant. ***, *p* < 0.001 and ****, *p* < 0.0001.

The differences we observed in the plasma pool proteomes on a protein identification level led us to investigate further how the lateral flow device impacts the detection of plasma proteins by mass spectrometry. For the sake of clinical relevancy, we focused on how the use of the device changed the plasma proteome in the “Good” and “Poor” NSCLC plasma pools (Figure 2). When “Good” NSCLC plasma applied to the BCD was compared to “Good” plasma prepared neat, there were 189 protein groups significantly associated with the device preparation and 207 protein groups significantly associated with the neat preparation (Figure 2A). In the “Poor” plasma pool, 195 proteins were significantly associated with the device preparation, while 231 protein groups were significantly associated with the neat preparation. Interestingly, the protein groups associated with either preparation method were largely consistent between the “Good” and “Poor” NSCLC plasma pools. Gene ontology (GO) enrichment analyses for molecular function, biological process and cellular compartment did not reveal any distinct biology to explain the association of either set of proteins with the neat or BCD preparations (Figure S1).

The proteins in each significantly associated set were then characterized for their physiochemical properties, including molecular weight, isoelectric point (pI), hydropathicity and instability index [33,34]. No significant differences were observed in molecular weight or pI between the neat and BCD preparations (Figure 2B,C). The average hydropathicity of neat-associated protein groups was −0.4837 compared to −0.3923 for the BCD-associated protein groups, indicating that neat-associated proteins were significantly more hydrophilic (Figure 2D). It has previously been shown that silica, as a component of the fiberglass membrane of the device, is known to interact with hydrophilic molecules [35]. These data suggest a potential mechanism by which the proteins enriched in the neat preparation are weakly adsorbed on the device membrane, resulting in their apparent depletion in the BCD preparation and enrichment in the neat [36]. The instability index, a measure of protein thermal stability in vitro based on dipeptide character, was significantly lower in the device-associated protein groups (39.7820) than in neat-associated protein groups (43.9376) (Figure 2E). A protein with an instability index greater than 40 is considered unstable, suggesting that the BCD enriches for more thermally stable proteoforms.

**Figure 3:**
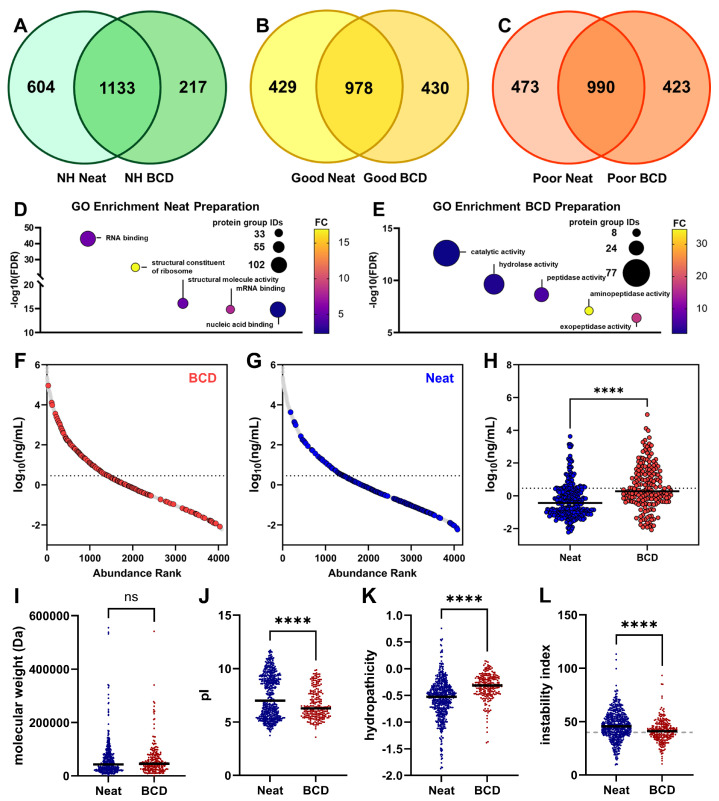
Preparation-specific protein groups. Venn diagrams for proteoforms detected in the neat and BCD preparations of (**A**) normal healthy, (**B**) NSCLC “Good” and (**C**) NSCLC “Poor” plasma pools. (**D**) GO enrichment analysis for molecular functions in the neat preparation-specific proteoforms across NH, NSCLC “Good” and NSCLC “Poor” plasma pools reveals conserved nucleic acid binding capacity. (**E**) GO enrichment analysis for the BCD-specific proteins showed shared enzymatic functions. Distribution of (**F**) protein groups detected exclusively with the use of the BCD (red) and (**G**) protein groups detected exclusively in the neat preparation (blue) compared to 4077 abundance-ranked proteins from the human plasma proteome project (HPPP, build 2021-07, grey). The dotted lines at 0.46 log_10_ (ng/mL) denote median abundance. (**H**) Protein groups detected exclusively in the BCD preparation were significantly more abundant in human plasma than protein groups detected only in the neat preparation. Physiochemical characterization of neat- (blue) and BCD-associated (red) protein groups, including (**I**) molecular weight, (**J**) pI, (**K**) hydropathicity and (**L**) instability index (stable:unstable demarcation of 40 marked in gray). FC, fold change; NH, normal healthy; ns, not significant. ****, *p* < 0.0001.

There were also proteins observed in the plasma from each preparation method that were not observed in the other preparations. There were 604, 429 and 473 protein groups in the NH, NSCLC “Good” and NSCLC “Poor” plasma pools prepared neat, respectively, that were not detected in the BCD preparation, while 217, 430 and 423 protein groups were observed in the NH, “Good” and “Poor” plasma prepared on the BCD that were not observed in the neat preparation (Figure 3A–C). To characterize these protein groups exclusive to a single preparation method, GO enrichment analysis for molecular function was performed on the combined preparation-specific protein sets from each of the plasma pools. Protein groups detected only in the neat preparation were highly enriched for nucleic acid binding activity, including RNA and mRNA binding and ribosomal structural constituents (Figure 3D). Fiberglass filters are used widely for the isolation of nucleic acids due to the strong interactions between silica and highly polar DNA and RNA molecules [37,38,39]. Like the proteoforms enriched in the neat preparation, we suspect that many of those detected exclusively in the neat preparation are adsorbed on the device membrane through their bound nucleic acids during the BCD preparation and thus are not detected. Protein groups detected only when the device was used were enriched for catalytic and enzymatic activities, which did not explain their exclusivity to the BCD preparation (Figure 3E). We then theorized that these were lower abundance protein groups that were only able to be detected after the device membrane depleted a portion of the plasma protein content; however, an abundance analysis revealed that BCD-specific proteoforms were significantly more abundant than neat-specific protein groups (Figure 3F–H).

As for the differentially enriched proteoforms, we further characterized the preparation-specific proteins for their physiochemical properties. No significant difference in molecular weight was observed between the BCD- and neat-specific protein groups (Figure 3I). BCD-exclusive protein groups were significantly more acidic than neat-specific proteoforms (Figure 3J). Again, the average hydropathicity for neat-specific proteins (−0.5621) was significantly lower than for BCD-specific proteins (−0.3315); however, the disparity between the two averages was greater than in the differentially enriched protein sets (Figure 3K). Following that trend, the average instability index for the device-exclusive proteins (41.2358) was again lower than for the neat-exclusive proteins (46.3022); however, in this case, both averages are considered unstable (Figure 3L).

### 3.3. Use of the BCD for a Clinical Immune Classifier

**Figure 4:**
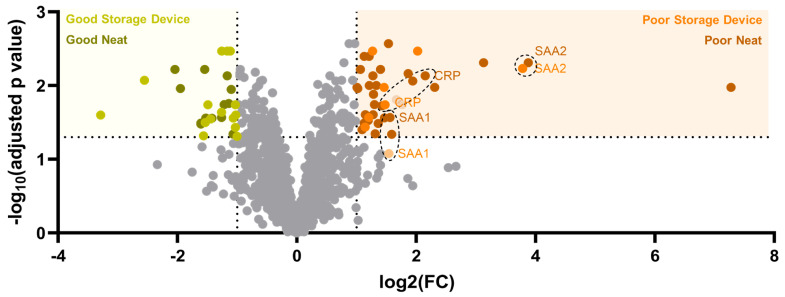
Proteins utilized by the immune classifier are not significantly retained on the BCD. Overlaid volcano plots of proteins significantly associated with NSCLC “Poor” (light orange, prepared on the BCD; dark orange, prepared neat) and NSCLC “Good” (light yellow, prepared on the BCD; dark yellow, prepared neat) plasma are shown. Serum amyloid A1 (SAA1), serum amyloid A2 (SAA2) and C-reactive protein (CRP) are enriched in the NSCLC “Poor” plasma pool regardless of preparation method.

Our data demonstrated that use of the device may impact some components of the plasma proteome and their availability for analytic studies. We then sought to test whether the BCD impacts the detection of proteins important for label stratification in a validated clinical immune classifier [40]. The classifier detects elevated expression of circulating serum amyloid A1, serum amyloid A2 and C-reactive protein in patients who are likely to benefit from combination immunotherapy and chemotherapy (NSCLC “Poor”) when compared to patients without elevated SAA1, SAA2 and CRP levels who are likely to benefit from single-agent immunotherapy (NSCLC “Good”) [22,23,24]. Use of the BCD did not significantly alter the detection of SAA2 or CRP and minimally impacted SAA1 (Figure 4). Whether prepared on the storage device or prepared neat, NSCLC “Poor” plasma had elevated levels of the relevant test proteins when compared to NSCLC “Good” plasma.

Overall, the BCD showed utility for a wide range of untargeted proteomic applications, from true discovery proteomic experiments to proteomic tests in clinical use. The device’s effect on certain protein analytes, including nucleic acid binders and particularly hydrophilic proteins, suggests that it should be evaluated for use on a case-by-case basis.

### 3.4. Targeted Proteomics with the Blood Collection Device

**Figure 5:**
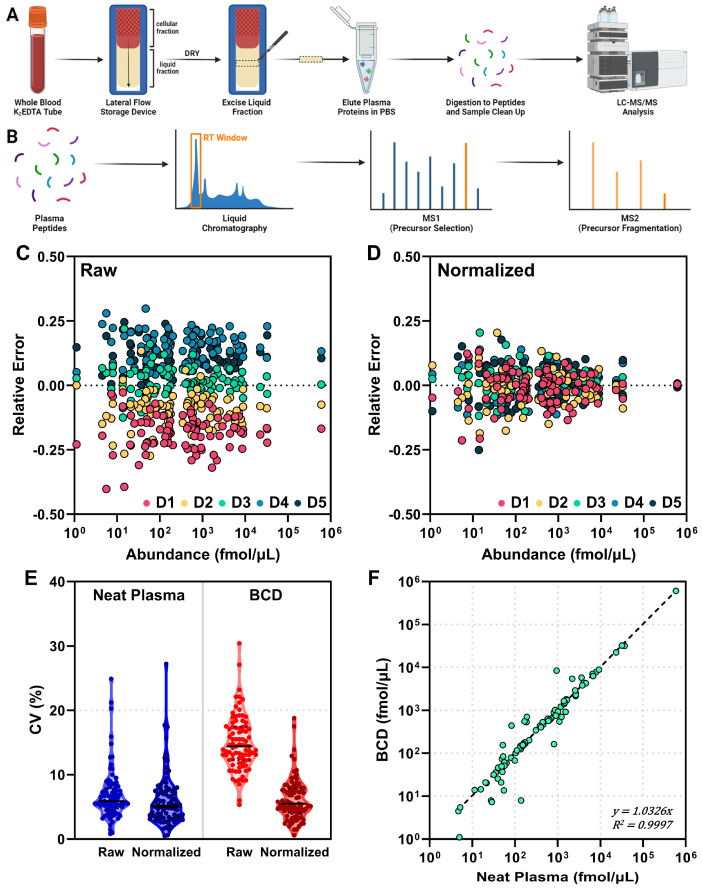
Correlation between targeted proteomic measurements on the BCD and in neat plasma. **(A**) Sample preparation workflow for plasma applied to the BCD. (**B**) Outline of targeted LC-MS/MS. (**C**) Raw (non-normalized) and (**D**) total protein normalized relative error (average measurement—single measurement/average measurement) for 89 proteins across five devices (D1, red; D2, orange; D3, teal; D4, light blue; and D5, dark blue). (**E**) Violin plots for raw and normalized protein measurement CV for the neat (blue) and BCD (red) preparations. The FDA 20% CV threshold is demarcated in gray. (**F**) Correlation plot for normalized neat and BCD measurements of 89 proteins, averaged across all devices. Dashed line, perfect correlation.

Clinical diagnostic or theranostic proteomic pipelines typically begin with untargeted discovery proteomic experiments, which identify potentially relevant disease or response markers, then culminate in the development of a targeted proteomic assay to specifically measure these analytes of interest [41,42]. To better understand how the BCD can support the full experimental spectrum in this pipeline, we assessed whether the use of the device would impact targeted, quantitative protein measurements. To this end, we performed targeted multiple-reaction monitoring (MRM) proteomic experiments on normal healthy plasma from a single donor that was applied to five devices and compared with the same plasma prepared neat five times without the use of the BCD (Figure 5A). We measured 89 total proteins, which spanned nearly five orders of magnitude in abundance (Figure 5B).

We first considered that the protein concentration of the lateral flow device eluate depends on the membrane area excised from the device, the volume of the extraction and the efficiency of protein recovery. To correlate protein levels between the device and the neat plasma, the same theoretical amount of total protein needed to be injected onto the column. Previous studies estimated the BCD to contain approximately 7.8 μL of plasma per cm^2^ of membrane [19], thus our injection volumes were calibrated using this factor.

After acquisition of the MRM data, an analysis of the relative error for each protein on a per-device basis showed a systematic offset for each device (Figure 5C). We then relied on a data normalization factor to correct the systematic deviation that occurred between devices. Many data normalization methods have been reported and implemented for MS datasets and should be considered based on specific assay requirements [43,44,45]. In this and all future experiments, a simple, single-point calibration based on the abundance of total protein was used for normalization according to Equation (1).

(1) $$R_{p,s}^{n}=R_{p,s}/\left(\frac{A_{s}}{A_{avg}}\right)$$

From Equation (1), a specimen-dependent correction factor is determined using the ratio of the normalization peptide in the specimen ($A_{s}$) to the average of the normalization peptide ratio over all five replicates ($A_{avg}$). The normalization correction factor is applied to all the response ratios in the specimen ($R_{p,s}$), where *p* is the peptide and *s* is the specimen, to obtain the normalized response ratio ($R_{p,s}^{n}$). Normalization using this routine substantially decreased the device-specific relative error and thus the overall offset (Figure 5D). Statistical analyses were performed to evaluate the percent coefficient of variation (CV) between the neat and BCD preparations (Figure 5E). Prior to normalization, the average CV for the BCD was 14.8%, which was approximately double the CV of the neat plasma preparation (6.8%). After applying Equation (1) for data normalization to both preparation methods, the average CV for the BCD decreased to 6.1%, which was nearly equivalent to the neat plasma (6.0%). The impact on the results was minimal, with correction factors ranging from 0.85 to 1.12. Before normalization, 9 of the 89 proteins in the BCD preparation were above the FDA-established criteria of a 20% CV threshold for biomarkers [46]. After normalization, all BCD proteins fell below this limit.

The normalized data show a strong correlation (R^2^ = 0.9997, Pearson Correlation = 0.9998) between the BCD and neat-prepared plasma for the 89 proteins measured across all devices (Figure 5F). Despite the strong concordance, there were proteins that showed deviation from the linear correlation between preparation methods. Proteins were observed above and below the linear correlation; however, the largest deviation appears in the low abundance range (<100 fmol/µL in plasma), with neat plasma showing several proteins having a higher relative abundance than the device. Taken together, these results support the utility of the BCD for acquiring targeted MRM proteomic data with a strong correlation to neat plasma.

### 3.5. Protein Abundance Measurements Between the BCD and Neat Plasma

**Figure 6:**
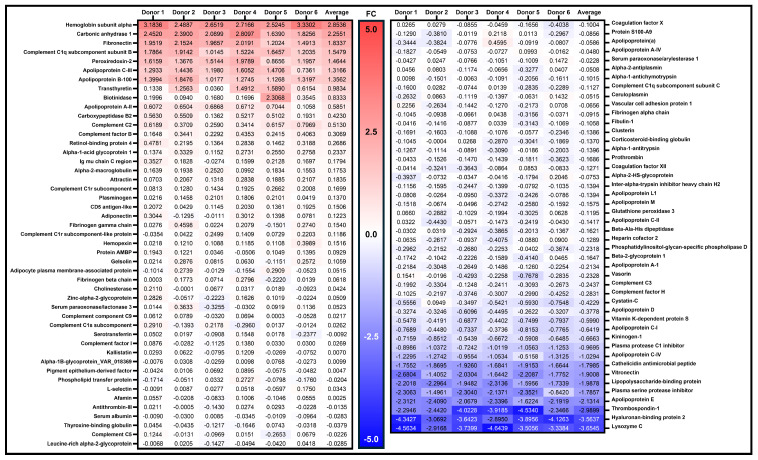
Heat map of normalized inter-donor protein abundance changes. The ratio of BCD/neat protein measurements across all six donors. Proteins highlighted in blue or red show a higher fold change in the BCD or in the neat preparations, respectively. The data were normalized to total protein content as previously described. FC, fold change.

The lateral flow device was further characterized by repeating the MRM-MS assay with five individual healthy donors (one device collected for each). MRM-MS was conducted for each donor with both the device and neat preparations of plasma. We also included data from the first experiment, thus analyzing a total of six donors. The protein abundances for the panel were again normalized to the total protein within each donor specimen. A heat map, shown in Figure 6, highlights the normalized BCD/neat ratios, or fold change differences, between the two methods. All but 12 of the 89 proteins had an acceptable range of ≤2-fold differences between the neat- and BCD-prepared plasma over all six donors. As examples, three proteins, hemoglobin alpha, carbonic anhydrase, and peroxiredoxin-2, are known to be in high abundance in RBCs. It is possible that variable levels of hemolysis among the donor specimens impacted the ratios measured for those proteins. Unlike the neat preparation method, the BCD utilizes a membrane in the separation step, and differential protein elution and/or blood cell lysis are potential variables among donors.

Concordance of protein measures post-recovery of plasma prepared neat or on the BCD was also assessed in the multi-donor experiment (n = 6 donors, one device each). When data from each donor are analyzed separately, the R^2^ values for the six donors ranged from 0.9994 to 0.9998 (Figure S2). The average across the six donors was R^2^ = 0.9996. Overall, the concordance in protein measured between the BCD and neat plasma was consistent over multiple donors, further supporting the device’s use in targeted assays.

### 3.6. Device Protein Migration Gradient

**Figure 7:**
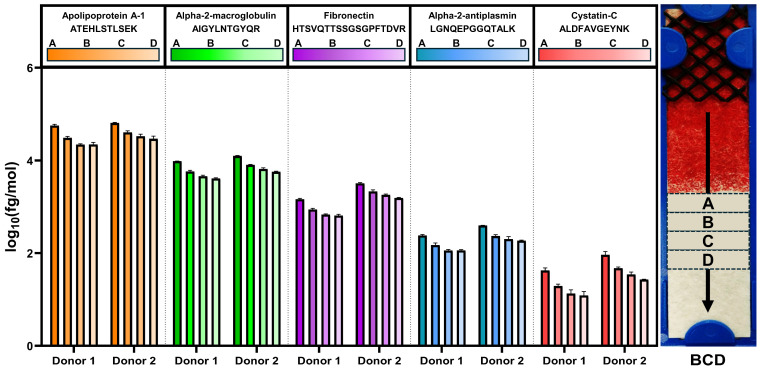
Representative protein concentrations in plasma recovered after lateral flow separation of whole blood. A representative image of a BCD post-whole blood separation is shown on the right, annotated with sections A–D, which were each 0.5 cm wide. Normalized concentrations for ATEHLSTLSEK, AIGYLNTGYQR, HTSVQTTSSGSGPFTDVR, LGNQEPGGQTALK and ALDFAVGEYNK peptides corresponding to apolipoprotein A-1, alpha-2-macroglobulin, fibronectin, alpha-2-antiplasmin and cystatin-C, respectively, are shown for each section for each donor. BCD, blood collection device.

With the separation of the RBC and liquid fractions, there is potential for a chromatographic effect, in which proteins migrate at different rates along the device based on their physical properties and interaction with the separation membrane. This could result in different relative protein concentrations across the device that would be position-dependent. To investigate the device for potential protein gradients, two donors with three devices each were tested. The workflow was modified such that four individual sections (0.5 cm long) were excised from each of the six devices, starting immediately following the red blood cell front. The first section was labeled A, and each subsequent 0.5 cm section was labeled B, C, and D, respectively. A schematic of how the device was sectioned is shown in Figure 7. Each section was analyzed individually by MRM-MS. Proteins were eluted from the separation device with 30 µL of PBS rather than the standard 80 μL to account for the smaller area of membrane to be analyzed.

The data for this study showed that the protein concentration did not change significantly between sections except for section A, the section immediately proximal to the RBC front. The general trend observed was the highest peptide abundance in section A, then decreasing in B, C, and D. Bar graphs from five representative peptides, ranging in concentration by four orders of magnitude, are shown in Figure 7. For each section, the concentrations were normalized to total protein, and then the replicates were averaged. We note that the replicates showed consistent trends across the sections. Both donors show similar trends in abundance and variability (CV) across the sections. Additionally, there were no proteins that were identified in section A that were not identified in section D. Furthermore, all peptides in the panel showed similar trends to the representatives shown.

The CVs observed were consistent and similar for each donor over the three replicates. Donor 1 had CVs for the sections that ranged from 7.9% to 9.7%. Donor 2 ranged from 7.5% to 9.2%. This indicates the flow of the blood was similar over the three replicates from each donor. The data were averaged between the two donors, the peptide concentrations were summed and averaged, and the standard deviation was assessed for all 89 peptides (Table 1). Section A had at times more than twice the recovered peptide concentration, leading to increased variability when compared to the other three sections. These data indicate that the first 0.5 cm section of the plasma fraction (the interface with red blood cells) should be excluded from extraction and downstream analyses. Thus, the most stable regions of recovery are in sections B, C and D. Additional data for the five representative peptides shown in Figure 7 can be found in Supplementary Table S1. We note that all peptides were measured in all sections. These results demonstrate that none of the proteins in the MRM-MS panel were being selectively fractionated within a membrane section after lateral flow. Together, these data suggest that the BCD eluate performs as well as neat plasma in targeted proteomic experiments.

## 4. Discussion

The ability of a novel whole blood collection device to support both discovery and highly multiplexed targeted proteomics was evaluated in this study. The device separates blood into solid cellular and liquid plasma fractions by simple lateral flow through a membrane at ambient temperatures [19]. After the specimen is dry, the device can be shipped and stored at ambient temperature while reducing the biohazards and costs associated with shipping whole blood or frozen plasma.

These advantages prompted additional characterization of the utility of the device for untargeted proteomic applications. We observed an increased number of protein IDs in the neat normal healthy donor pool when compared to the NSCLC “Good” and “Poor” pools, potentially due to differential sourcing of the NSCLC and NH plasmas. Alternatively, disease-related fluctuations in the NSCLC plasma proteome could account for this difference. Use of the BCD dramatically reduced variability in protein group identification in normal healthy and NSCLC plasma pools when compared to the same plasmas prepared neat without the device (Figure 1). It is possible that by reducing the overall protein content of the sample, the BCD stabilizes variation in the remaining proteoforms by preventing oversaturation of the MS detector [47].

As expected, the BCD does impact the detectable plasma proteome. Proteins that were enriched in the neat preparation, those partially depleted after preparation on the BCD, were significantly more hydrophilic than proteins enriched in the BCD preparation (Figure 2). The reversible adsorption of hydrophilic proteins on silica-based fiberglass membranes like those in the device has been well characterized and may explain a mechanism by which these proteins are transiently retained on the device and thus partially depleted from the sample [48]. Conversely, the significantly more hydrophobic character of the BCD-associated proteins may suggest a mechanism by which these proteins are enriched in the BCD preparation. It is possible that these proteins are transiently absorbed on the hydrophobic surface of the polypropylene tube used to store the neat plasma and are thus partially depleted from the neat dataset. Reversible adsorption of proteins on solid polymer surfaces is a previously documented phenomenon that is strongly influenced by the hydrophobic character of the adsorbed molecule [49]. These results highlight the important influence that laboratory consumables can have on proteomic experiments.

Many proteins were detected exclusively in each of the sample preparation methods (Figure 3). Protein groups absent from the BCD preparation and thus detected only in neat plasma were highly enriched for nucleic acid binding functions. It is likely that these proteins are irreversibly adsorbed on the device membrane through their bound nucleic acids and thus are only detected in the neat preparation. The mechanism for the strong interaction between polar nucleic acids and silica fiberglass has been well characterized and fiberglass membrane filters are frequently used to isolate nucleic acids from their biological matrices [50,51,52]. Importantly, these findings suggest that free nucleic acids in these plasma samples may also be adsorbed onto the membrane and thus posit the potential to use the BCD for cell-free DNA (cfDNA), cell-free RNA (cfRNA) and circulating tumor DNA (ctDNA) isolation or analysis. It is likely that a combination of characteristics, predominantly hydrophobicity but to a lesser extent pI and protein stability, result in the irreversible adsorption of the BCD-exclusive proteins onto the inner surface of the polypropylene tube used in the neat preparation or the aggregation of hydrophobic proteins through agitation at the liquid–air junction in the tube [53]. The complex adsorption and aggregation dynamics of proteins in hydrophobic polypropylene containers are understood to be influenced by a variety of physiochemical protein properties [54]. Even the use of treated plastics for proteomics like those employed in this study has been shown to not fully attenuate protein loss to these phenomena [55]. We have therefore identified another advantageous attribute of the BCD in that it can mitigate the effects of protein loss by adsorption at the liquid–solid interface and aggregation at the liquid–air interface in laboratory consumables.

Given the potential of the device to retain certain classes of protein, we assessed its impact on the clinical immune classifier developed by our group (Figure 4). Use of the BCD had a minimal impact on the detection of NSCLC “Poor”-associated proteins SAA1, SAA2 and CRP. These proteins remained elevated in the “Poor” plasma pool relative to the “Good” plasma pool regardless of whether the plasma was prepared on the BCD or neat. These data suggest that many proteomic assays could benefit from the use of the device; however, such assays should be assessed on a case-by-case basis to evaluate the impact of the BCD on the analyte of interest.

For additional clinical relevancy, the lateral flow device was then investigated for use in quantitative proteomic applications. Concordance between measures of protein recovered from the BCD and neat plasma preparation was consistent across intra-donor replicates (Figure 5). Of note was the potential benefit of normalizing the results for the individual peptides measured to the total protein measured. Prior to normalization, the BCD data set had ~2-fold higher CVs than the centrifuged plasma method, and 9 of the 89 proteins had CVs above the 20% threshold criteria set out by the FDA [46]. Normalization corrected both metrics with minimal impact on the results. Additional normalization routines, including normalizing to partial or total ion current, are being investigated [56].

An analysis of inter-donor performance of the BCD as compared to neat preparation demonstrated that more than 80% of the protein panel were in excellent agreement, with normalized BCD/Plasma abundance ratios ≤2 (Figure 6 and Figure S2). However, a subset of proteins consistently showed a higher abundance ratio (>2-fold) in either the BCD or centrifuged plasma method across all six individual donors. It is noteworthy that several of these proteins exhibiting the highest fold difference in the device eluate, such as hemoglobin, carbonic anhydrase 1, and peroxireddoxin-2, are known to be in high abundance in RBCs [57]. The blood separation process, through friction against glass fibers in the device membrane, can result in the lysis of more RBCs than when centrifuging whole blood [58,59]. While the BCD removes most RBCs from the dried plasma spot destined for analysis, applications sensitive to the presence of hemoglobin may need to be further processed for use with the device.

The protein panel used in the targeted experiments spans nearly five orders of magnitude, yet the normalization method corrected variations in proteins across the entire dynamic range. These data indicate that non-biased separation and extraction are possible with lateral flow devices such as the one in this study. The greatest observed variability was attributable to differences at the proximal interface with the red blood cells (Figure 7). An analysis of protein gradients in the membrane demonstrated that all 89 analyzed proteins were detected in each of the 4-membrane quadrants spanning the RBC interface to 2 cm from the edge of the separation membrane. Furthermore, the mobility trend was similar for all analyzed proteins, with about twice as much protein abundance closer to the red blood cell interface (Table 1). This may contribute to the variation observed in the raw BCD data; however, excising a larger membrane section that avoids the first 0.5 cm section at the RBC interface would largely minimize this effect.

The data presented herein further establish the analytic utility of the BCD as a robust and reproducible tool for discovery and quantitative plasma proteomics and add to clinical utility data previously published for the device [19]. The protein analytes recovered from the device can be analyzed both in untargeted and highly multiplexed targeted MS assays. The device has several key improvements over centrifuged plasma, including ambient temperature transport, longer specimen stability, no centrifuge, lower biohazard risks and ease of use. Thus, the BCD can support emerging areas of critical need like blood sample collection and transport in remote locations.

## 5. Conclusions

Our findings show that blood collection devices utilizing lateral flow methods can present the broader clinical proteomic community with a safer, low-cost and simple-to-use blood separation device. The BCD investigated herein is proven to be a robust solution for the transport and storage of plasma proteomics, with advantages over traditional plasma preparation methods such as ambient temperature shipping and reduced biohazard risk. Furthermore, we show that the plasma proteins recovered from the BCD can be used in both targeted and untargeted proteomics MS approaches. These advantages make the BCD an attractive tool for the storage and transport of analytes for clinical applications.

## 6. Patents

US Patent Number 10422729.

## Figures and Tables

**Table 1:** Summary statistics for the sections of the BCD across 89 peptides.

Section	Sum (fmol/μL)	Average (fmol/μL)	Standard Deviation (fmol/μL)
A	1,644,948	18,691	516
B	1,040,229	11,820	313
C	879,382	9991	350
D	800,815	9099	258

## Data Availability

No additional data reports.

## Supplementary Materials

The following supporting information can be downloaded at: https://www.mdpi.com/article/10.3390/biomedicines12102318/s1, Figure S1: Gene ontology enrichment analyses for neat- and BCD-associated plasma proteins; Figure S2: Inter-donor concordance. Table S1: Proteins recovered from lateral flow-separated sections of the BCD. Supplementary data: raw untargeted data, reliably detected proteins, and MRM data.
