# Supplementary material for: Use of a Novel Whole Blood Separation and Transport Device for Targeted and Untargeted Proteomics
Source: Biomedicines. 2024 Oct 11;12(10):2318. doi: 10.3390/biomedicines12102318 (PMC11504527; doi:10.3390/biomedicines12102318)
Supplement: Supplementary file 1 [file biomedicines-12-02318-s001.zip › biomedicines-3216564-supplymentary-word.pdf]

Supplementary Figures and Tables

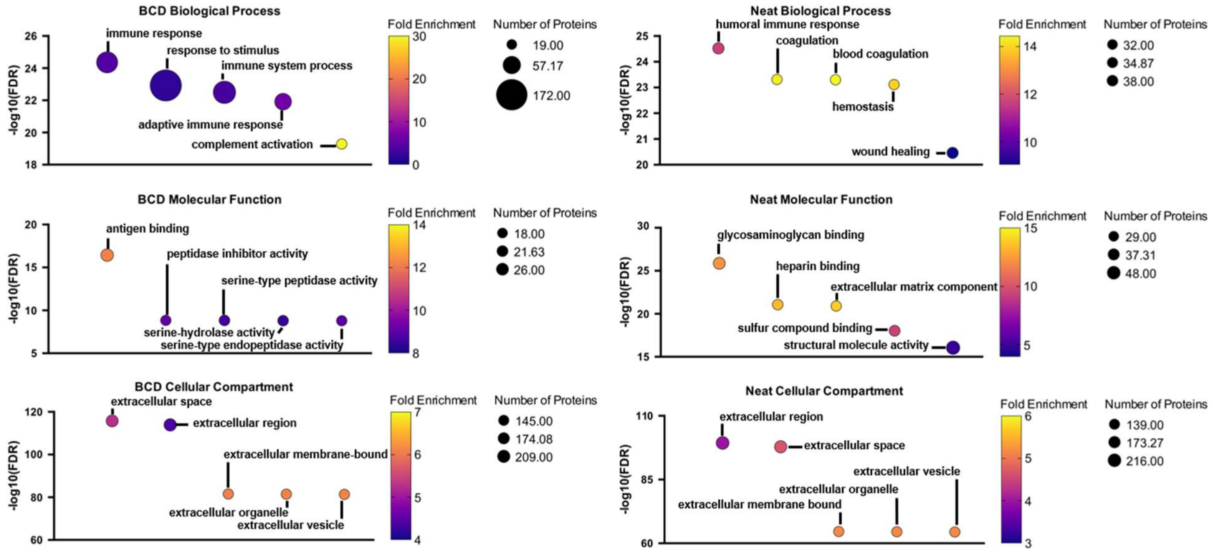

Figure S1. Gene Ontology Enrichment Analyses for Neat and BCD-Associated Plasma Proteins. *Gene Ontology enrichment analyses for biological processes, molecular functions and cellular compartments were performed on device and neat-associated protein groups from combined NSCLC "Good" and "Poor" plasma pools.*

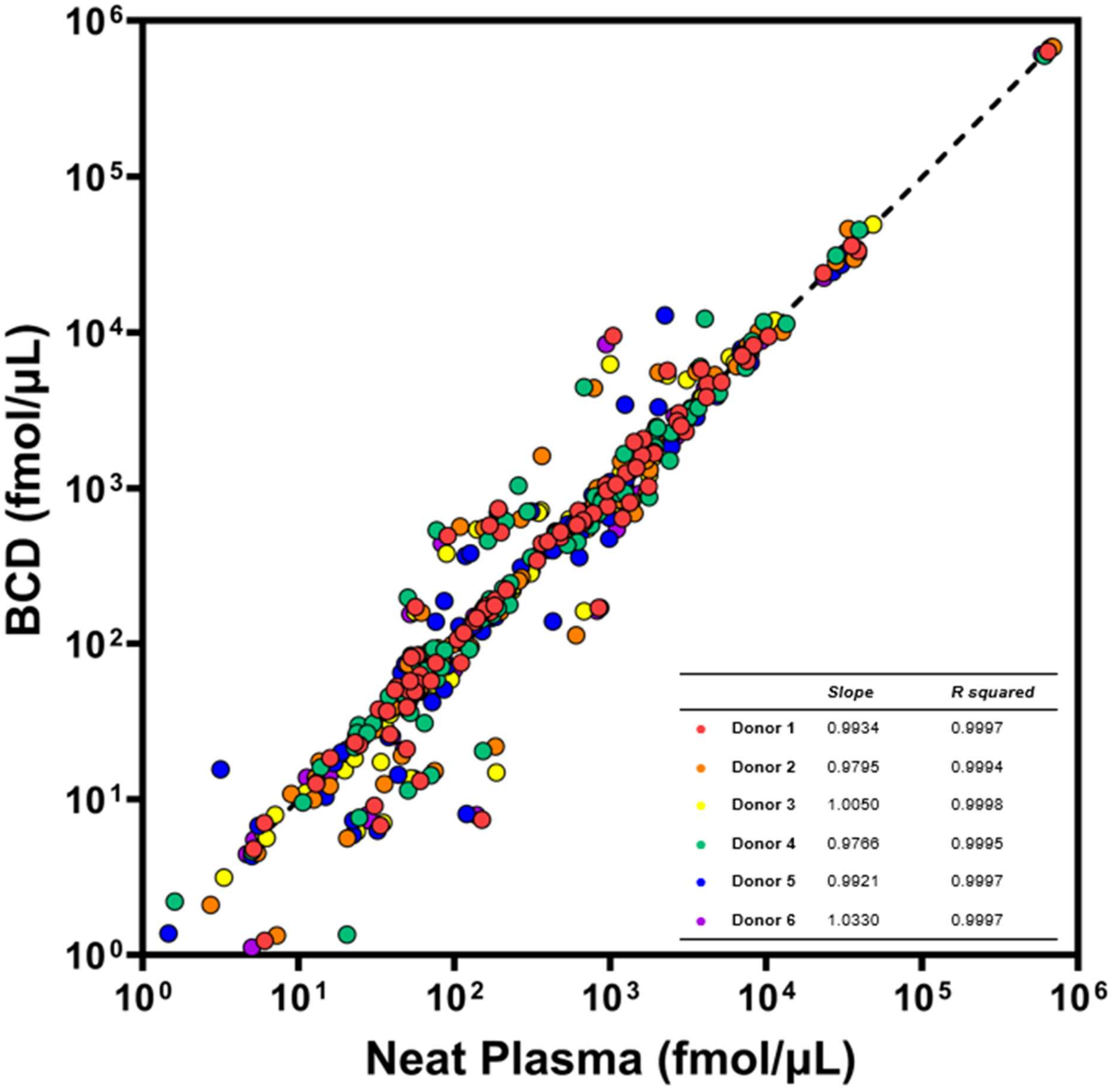

Figure S2. Inter-donor Concordance. *Correlation plot showing normalized neat and BCD measurements of 89 proteins across 6 individual donors.*

Table S1. Proteins Recovered From Lateral Flow-Separated Sections of the BCD.

Donor 1	***ATEHLSTLSEK***							***Apolipoprotein A-1***
	Section	*Rep1*	*Rep2*	*Rep3*	*Sum*	*Average*	*SD*	*CV (%)*
	A	60648	54023	56090	170761	56920	3390	6.0
	B	33294	30111	28758	92163	30721	2329	7.6
	C	21738	23160	21585	66473	22158	871	3.9
	D	20235	21617	24972	66823	22274	2436	10.9
	AIGYLNTGYQR							***Alpha-2-macroglobulin***
	Section	*Rep1*	*Rep2*	*Rep3*	*Sum*	*Average*	*SD*	*CV (%)*
	A	9699	9716	9632	29048	9683	44	0.5
	B	6037	5918	5433	17387	5796	320	5.5
	C	4773	4657	4281	13711	4570	257	5.6
	D	4147	3920	4210	12277	4092	152	3.7
	HTSVQTTSSGSGPFTDVR							***Fibronectin***
	Section	*Rep1*	*Rep2*	*Rep3*	*Sum*	*Average*	*SD*	*CV (%)*
	A	1457	1521	1392	4370	1457	65	4.4
	B	915	826	900	2642	881	48	5.4
	C	706	677	661	2043	681	23	3.4
	D	675	603	670	1949	650	40	6.2
	LGNQEPGGQTALK							***Alpha-2-antiplasmin***
	Section	*Rep1*	*Rep2*	*Rep3*	*Sum*	*Average*	*SD*	*CV (%)*
	A	239	232	253	724	241	10	4.2
	B	164	132	154	450	150	16	10.7
	C	118	117	108	343	114	5	4.8
	D	112	120	113	344	115	4	3.8
	ALDFAVGEYNK							***Cystatin-C***
	Section	*Rep1*	*Rep2*	*Rep3*	*Sum*	*Average*	*SD*	*CV (%)*
	A	46	45	37	128	43	5	11.7
	B	21	21	18	59	20	2	8.7
	C	12	17	13	41	14	3	19.1
	D	11	15	11	37	12	3	20.6
Donor 2	***ATEHLSTLSEK***							***Apolipoprotein A-1***
	Section	*Rep1*	*Rep2*	*Rep3*	*Sum*	*Average*	*SD*	*CV (%)*
	A	64208	63139	66207	193554	64518	1557	2.4
	B	42828	36858	41866	121551	40517	3205	7.9
	C	29792	33405	36917	100114	33371	3562	10.7
	D	25547	30194	32846	88586	29529	3695	12.5
	AIGYLNTGYQR							***Alpha-2-macroglobulin***
	Section	*Rep1*	*Rep2*	*Rep3*	*Sum*	*Average*	*SD*	*CV (%)*
	A	12777	12525	12260	37562	12521	258	2.1
	B	7853	8119	8181	24153	8051	174	2.2
	C	6423	6295	7000	19718	6573	375	5.7
	D	5634	5647	5899	17181	5727	149	2.6

HTSVQTTSSGSGPFTDVR							***Fibronectin***
Section	*Rep1*	*Rep2*	*Rep3*	*Sum*	*Average*	*SD*	*CV (%)*
A	3186	3333	3114	9633	3211	111	3.5
B	2292	2198	1965	6456	2152	168	7.8
C	1843	1758	1875	5476	1825	61	3.3
D	1600	1529	1536	4666	1555	39	2.5
LGNQEPGGQTALK							***Alpha-2-antiplasmin***
Section	*Rep1*	*Rep2*	*Rep3*	*Sum*	*Average*	*SD*	*CV (%)*
A	400	393	398	1191	397	4	0.9
B	245	242	218	704	235	15	6.3
C	229	183	197	610	203	24	11.6
D	192	182	186	560	187	5	2.6
ALDFAVGEYNK							***Cystatin-C***
Section	*Rep1*	*Rep2*	*Rep3*	*Sum*	*Average*	*SD*	*CV (%)*
A	101	78	102	281	94	14	14.7
B	51	46	47	143	48	3	5.5
C	37	31	38	105	35	4	11.0
D	27	27	28	81	27	0	1.3

Data shown for 5 representative peptides with triplicate measures over four sections (A – D) and for two individual donors. Replicate, sum, average and SD measured in fmol/µL. CV, coefficient of variation; Rep, replicate; SD, standard deviation.
